# Bioactive conformational generation of small molecules: A comparative analysis between force-field and multiple empirical criteria based methods

**DOI:** 10.1186/1471-2105-11-545

**Published:** 2010-11-04

**Authors:** Fang Bai, Xiaofeng Liu, Jiabo Li, Haoyun Zhang, Hualiang Jiang, Xicheng Wang, Honglin Li

**Affiliations:** 1 Faculty of Chemical, Environmental and Biological Science and Technology, Dalian University of Technology, Dalian 116023, PR China; 2 Department of Engineering Mechanics, State Key Laboratory of Structural Analysis for Industrial Equipment, Dalian University of Technology, Dalian 116023, PR China; 3 State Key Laboratory of Bioreactor Engineering & Shanghai Key Laboratory of Chemical Biology, School of Pharmacy, East China University of Science and Technology, Shanghai 200237, PR China; 4 Drug Discovery and Design Center, State Key Laboratory of Drug Research, Shanghai Institute of Materia Medica, Chinese Academy of Sciences, Shanghai 201203, PR China; 5 Accelrys Inc., 10188 Telesis Court, San Diego, California 92121, USA

## Abstract

**Background:**

Conformational sampling for small molecules plays an essential role in drug discovery research pipeline. Based on multi-objective evolution algorithm (MOEA), we have developed a conformational generation method called Cyndi in the previous study. In this work, in addition to Tripos force field in the previous version, Cyndi was updated by incorporation of MMFF94 force field to assess the conformational energy more rationally. With two force fields against a larger dataset of 742 bioactive conformations of small ligands extracted from PDB, a comparative analysis was performed between pure force field based method (FFBM) and multiple empirical criteria based method (MECBM) hybrided with different force fields.

**Results:**

Our analysis reveals that incorporating multiple empirical rules can significantly improve the accuracy of conformational generation. MECBM, which takes both empirical and force field criteria as the objective functions, can reproduce about 54% (within 1Å RMSD) of the bioactive conformations in the 742-molecule testset, much higher than that of pure force field method (FFBM, about 37%). On the other hand, MECBM achieved a more complete and efficient sampling of the conformational space because the average size of unique conformations ensemble per molecule is about 6 times larger than that of FFBM, while the time scale for conformational generation is nearly the same as FFBM. Furthermore, as a complementary comparison study between the methods with and without empirical biases, we also tested the performance of the three conformational generation methods in MacroModel in combination with different force fields. Compared with the methods in MacroModel, MECBM is more competitive in retrieving the bioactive conformations in light of accuracy but has much lower computational cost.

**Conclusions:**

By incorporating different energy terms with several empirical criteria, the MECBM method can produce more reasonable conformational ensemble with high accuracy but approximately the same computational cost in comparison with FFBM method. Our analysis also reveals that the performance of conformational generation is irrelevant to the types of force field adopted in characterization of conformational accessibility. Moreover, post energy minimization is not necessary and may even undermine the diversity of conformational ensemble. All the results guide us to explore more empirical criteria like geometric restraints during the conformational process, which may improve the performance of conformational generation in combination with energetic accessibility, regardless of force field types adopted.

## Background

Conformational sampling, as an essential part of the molecular modelling process, is an important prerequisite for many applications in computational chemistry[1-4], especially in drug discovery research[5-9]. It has been a research hotspot in the last decades and many computational algorithms have appeared in succession recently[3,5,10-17]. Generally, these methods can be classified into two main categories: systematic methods and stochastic methods. Systematic methods exhaustively enumerate all possible torsions at certain discrete intervals[15], therefore such approach is usually limited to small molecules and becomes inapplicable for very flexible molecules due to the combinatorial explosion[15,18,19]. To overcome the combinatorial difficulty of systematic search algorithms, many programs, such as CAESAR[15], OMEGA[20] and CONAN[21] have adopted the divide-and-conquer strategy to divide the molecule into small pieces and then assemble conformations of the whole molecules from small fragments. However, some of these methods may still be insufficient to restrain the space of accessible conformers due to the limitation of the predefined templates (or other heuristics)[22]. In addition, the more common ways to avoid combinatorial explosion are the stochastic methods, such as random search[23] and molecular dynamics[24], which rely on certain random perturbations but usually still spend considerable computational time on energy minimization Therefore, developing a fast and reliable conformational generation engine is still a challenging task[10,11].

Recently, many observations have revealed that the bioactive conformation of a flexible molecule does not correspond to its global energy minimum or even some local minima on the potential energy surface in most cases [25-27]. In essence, flexible molecules often exhibit several conformations or geometries with nearly equal energies, and the one with favoured inter- and intra-interactions is adopted under given conditions, as stated by G. Klebe et.al., reliance on just the crystal structures of a ligand itself may not be an infallible or reliable indicator of biologically active conformation[28]. In addition, the standard algorithms for energy minimization in the commonly available molecular mechanics programs are mainly limited to downhill-energy search[29], so some potential conformational space is unavoidable to be neglected unexpectedly. For drug discovery and design applications, such as pharmacophore modelling, we usually have limited knowledge about the binding conformation of the potential drug. All these remind us to consider geometric diversity of conformations or other unrevealed criteria in addition to energy threshold simultaneously when searching conformational space[2,5].

There are some methods to promote geometric diversity, such as poling algorithm [30] used in Catalyst. Recently, we have introduced a new conformational sampling method named Cyndi and its detailed algorithm has been discussed in the previous work [2]. Cyndi is based on the multiple objective evolution algorithm (MOEA) and adopts different energy terms and geometric objectives to rationally explore in the conformational space. Therefore, it can ensure that the generated conformation ensemble simultaneously meets multiple criteria, such as low energy and geometric diversity instead of only concentrating on one of them[2]. This gives rise to two requirements on the outstanding prediction of conformational energies and good prediction of molecular geometries when modelling the conformations. Force fields, as the most common predictors for energies and geometries of molecules, vary in which they are developed to be applied to different aspects of bioorganic chemistry with specific sets of data[31]. Therefore it is necessary to explore the effect against different force fields adopted on the quality of conformational ensembles during the process of conformational analysis.

In this analysis, Cyndi has been updated by implementation of MMFF94 force field in addition to the default Tripos force field in previous version. With two force fields adopted in Cyndi, a detailed comparative analysis between force field based method (FFBM) and multiple empirical criteria based method (MECBM) is performed without force-field type bias. Furthermore, a larger dataset of 742 ligands with their bioactive conformations retrieved from PDB is used to validate the performance of both FFBM and MECBM in terms of conformational searching speed and biologically relevant conformations reproduction. In addition, the impact of energy minimization on conformational ensemble quality is also examined in this study. To have more confidence in our findings, we extend this literature with a further comparison on multiple conformational sampling techniques available in Cyndi and MacroModel integrated in Maestro V7.5 (Schrodinger Inc.).

## Methods

### Validation Dataset

The dataset was originally used for the validation work of CAESAR algorithm[15]. In this work, 15 molecules were removed firstly from the original 918 molecules, which contain atoms other than organic elements C, O, N, S, F, Cl, Br, P and H. Secondly, since the PDB data itself has a lot of errors, the molecules were therefore manually checked and the confusing ones whose structures in RCSB website and PDB files are inconsistent were also removed. After removing those structurally duplicated molecules, 746 molecules were left and their protonation states were assigned to them using Pipeline Pilot v6.0 (Accelrys, Inc.) in default condition (pH = 7.3). The errors in valence/charge assignment were manually corrected via visual inspection. Since the dependence of the conformational ensemble quality on input geometry is a challenge in conformational searching, Corina v3.1[32] was used to generate new 3D conformations for the molecules in the test set as the input conformations in this study. It should be pointed out that Corina failed to generate 3D conformations for 4 out of 746 molecules; therefore, the final dataset involves 742 molecules and is available in Additional File 1.

### Conformational Sampling with FFBM

In both FFBM and MECBM approaches, the bond lengths and bond angles are fixed as input. Only two terms, namely Van der Waals (VDW) and torsion energies are used as the force field objective functions in Cyndi for FFBM case. The numbers of population and generation during the evolution process were set to 200, and the epsilon values for the two objectives (VDW and torsion energies) in the epsilon multi-objective evolution algorithm (*ε*-MOEA)[33] were set to 5.0 kcal/mol and 3.0 kcal/mol, respectively. The maximum number of the conformations to be generated for each molecule was set to 600, and the maximum iteration for the optional energy minimization was set to 100. All the other parameters were set as default values in Cyndi. Any structure with energy more than 20 kcal/mol above the lowest conformation energy ever identified was discarded.

### Conformational Sampling with MECBM

For MECBM, two more objective functions, 1) geometric dissimilarity (GD) between each new conformation and the input conformation, and 2) the gyration radius (GR) for each conformation, which are directly related to conformation geometries were used. The epsilon values for these two objectives were set as 0.4 Å and 0.1 Å, respectively. In order to make a fair comparison, all other related parameters were set to the same values as in FFBM.

### Energy minimization for FFBM and MECBM

To access the impact of minimization, a series of protocols were repeated using identical settings but with the additional minimization procedure (denoted as FFBM_MIN or NECBM_MIN). By using the full force field excluding electrostatic energy term[2,5,27,34,35] and conjugate-gradient algorithm, each initially generated raw conformation was minimized with the same or the other force field complied with mixed force field strategy (in this way, the molecular conformational space could be explored with one force field and the raw conformations were post-refined with the other one) up to 100 steps or to the final convergence (0.01 kcal·mo^l-1^·Å^-1^).

### Conformational Sampling with MacroModel

Several algorithms for conformational analysis are implemented in MacroModel. In this work, Serial low-mode sampling (LMCS)[36], Serial torsional sampling (MCMM)[37] and serial torsional/low-mode sampling were used for the comparison. The torsional sampling option of MCMM and torsional/low-mode was set as "Extended" mode. Any structure with energy higher than the same energy threshold value as in FFBM and MECBM would be discarded. Default values were used for the other parameters related to conformational sampling. After the raw conformers were analyzed, MMFFs force field and OPLS-2005 force field which are available in MacroModel, were selected to minimize these raw conformers in vacuum respectively. The energy minimization was carried out using the Steepest Descent (SD) method, the minimization step was set as 100 and final convergence was set as 0.01 kcal·mol^-1^·Å^-1^.

### Tools and Benchmarks for Conformational Analysis

The performance of conformational generation methods was evaluated by the quality and efficiency to reproduce the bioactive conformations. The ability to retrieve the bioactive conformation at different levels of sampling can be measured by root mean square deviation (RMSD) between the best fitting conformation in the conformation ensemble and the corresponding bioactive conformation. Moreover, the distributions of conformational energies and gyration radius of the conformation ensemble were also studied in detail. The efficiency was measured by the computational time consumed for each test. All the test runs were performed on Intel(R) Xeon(R) CPU E7420 (2.13 GHz) and the quality and efficiency analyses were performed with an in-house developed protocol using Pipeline Pilot v6.0, which is also available in Additional File 2.

## Results

### Performance of the conformational search methods

**Figure 1:**
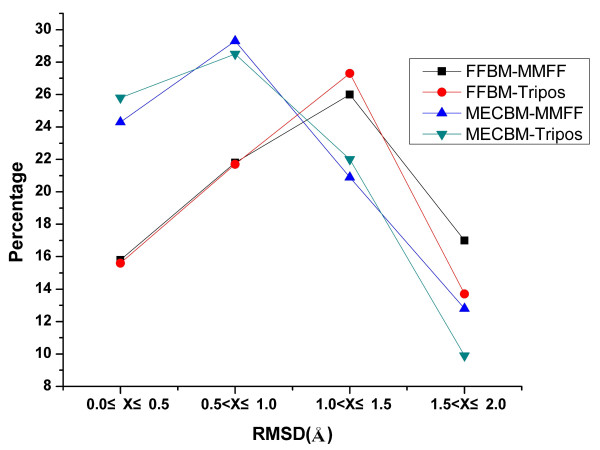
**Cumulative distribution of RMSD between the bioactive conformers and their best fitted generated conformers for FFBM and MECBM with either MMFF94 or Tripos force field**.

To assess the performance of FFBM and MECBM, the cumulative distributions of RMSDs between the bioactive conformers and their best fitting conformers in the generated conformer ensembles for each conformational search methods are presented in Figure 1 and Table 1. The figure shows the percentage of ligands for which there is at least one conformation identified within 0.5, 1.0, 1.5 and 2.0 Å RMSD intervals to the corresponding crystal structure by each searching protocol under evaluation. About 25% of the bioactive conformations can be reproduced by MECBM with RMSD falling within 0-0.5 Å RMSD interval, while only about 15% of the bioactive conformations can be reproduced by FFBM with RMSD falling within the same interval. For 0.5-1 Å RMSD interval, the recovery rate of MECBM amounts to 29%, while the counterpart of FFBM is lower than 25%, which indicate that adding two more geometric objectives in the conformation sampling can greatly improve the reproduced ratio of bioactive conformations.

**Table 1:** Statistics for the Different Conformational Search Protocols

Method	Force Field	Bioactive Conformation Reproduction Rate (%)				No. Conf. per mol	CPU time per mol(s)
				
		≤ 0.5Å	≤ 1Å	≤ 1.5Å	≤ 2.0Å		
FFBM	MMFF94	15.8	37.6	63.6	80.6	6	0.3
	Tripos	15.6	37.3	64.6	78.3	6	0.3
FFBM_MIN	MMFF94	15.5	39.8	63.5	81.1	6	1.3
	Tripos	15.6	40.6	65.6	80.5	7	1.3
MECBM	MMFF94	24.3	52.6	74.5	87.3	34	0.4
	Tripos	25.8	54.3	76.3	86.2	35	0.4
MECBM_MIN	MMFF94	26.5	54.1	77.8	87.2	42	5.2
	Tripos	26.3	54.3	75.7	87.2	43	5.2
LMCS	MMFFs	24.5	49.5	69.8	83.4	131	112.2
	OPLS-2005	24.4	49.1	71.1	84.9	165	106.8
MCMM	MMFFs	43.6	71.8	86.5	94.3	523	41.7
	OPLS-2005	42.1	70.8	89.4	94.2	567	41.8
Torsional/Low-mode	MMFFs	35.7	68.4	86.1	93.4	306	132.0
	OPLS-2005	37.9	68.5	86.6	93.5	354	125.9

Another interesting observation in the tests is that the force field type does not seem to play an important role in both MECBM and FFBM. In other words, MMFF94, whose efficiency in structure optimization has ever been proved[36], seems to be incapable of improving the quality of the conformers generated by MECBM or FFBM as expected. For FFBM case, the difference in retrieval rate with MMFF94 force field and Tripos force field is only 0.3% within 1 Å RMSD interval (37.6% and 37.3%), and for MECBM case, the retrieval rate with MMFF94 force field seems slightly lower than that with Tripos force field at a lower RMSD cutoff (24.3% and 25.8%, within 0.5 Å RMSD) (Table 1 and Figure 1). At a higher RMSD cutoff within 2.0Å, MMFF94 force field also seems to have a slightly positive impact (87.3% for MMFF94 and 86.2% for Tripos).

**Figure 2:**
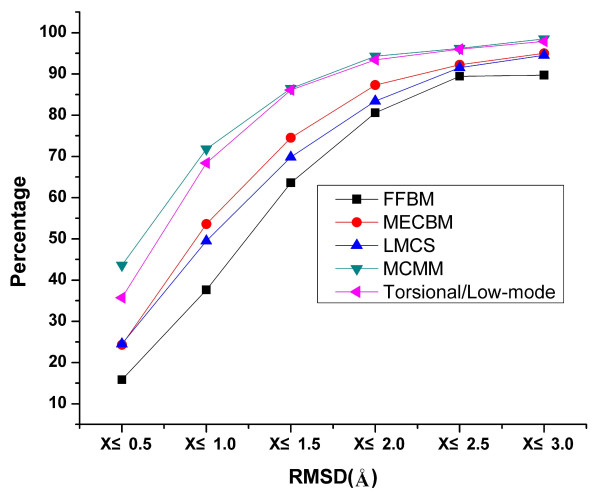
**Distribution of minimum RMSD between the bioactive conformations and their best fitting conformations for the five conformational sampling methods (FFBM and MECBM in Cyndi, LMCS, MCMM and torsional/low-mode sampling)**. Both FFBM and MECBM are cooperated with MMFF94 force field, all the three methods in MacroModel are cooperated with MMFFs force field.

The effectiveness of the other three conformational search methods in MacroModel was assessed mainly using three different metrics: the ability to identify the bioactive conformers, the average CPU time consumed for each molecule and the average size of conformational ensemble. The impact of force field type on these methods was also considered in this section. To simplify our comparative analysis, the potential dependence of our results on the particular force field used for cooperating with the three methods would be firstly considered. For LMCS, MCMM and torsional/low-mode, the differences in the retrieval rate (within 2Å) with MMFFs and OPLS-2005 are only 1.5% (83.4% for MMFFs and 84.9% for OPLS-2005), 0.1% (94.3% for MMFFs and 94.2% for OPLS-2005) and 0.1% (93.4% for MMFFs and 93.5% for OPLS-2005) respectively. These results came to the conclusion that the conformational search methods didn't thoroughly depend on the force field type, and the following comparative analysis would be focused on the abilities of MacroModel and Cyndi with the same MMFF94 force field (Figure 2). The recovery rates by MCMM and torsional/low-mode outperform any other remaining methods, and MECBM results in a better quality of conformational ensemble than LMCS in MacroModel, undoubtedly, FFBM which only considers the Van der Waals (VDW) and torsion energies of force field terms performed the worst for conformational generation.

**Figure 3:**
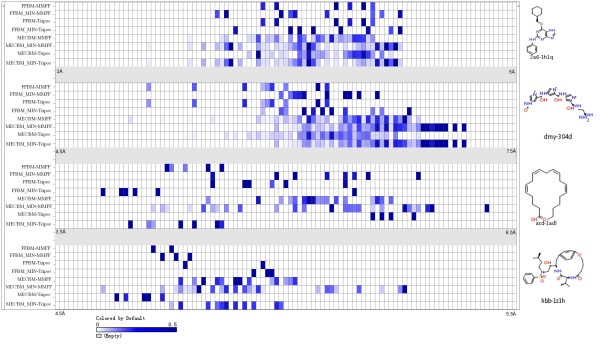
**Heat map showing the distribution of gyration radius of the conformers obtained by each test protocols designed for FFBM and MECBM**. The names of the different protocols are defined as *M-F *where *M *is the method chosen to execute conformational sampling job, *F *is the type of force field adopted. The scale of each molecule panel covers the full range of gyration radius encountered across all conformations within the energy threshold window (20 kcal/mol above the global lowest energy identified by each test run). The intensity of the cell colour is proportional to the fractions of conformations that fall in each bin.

**Figure 4:**
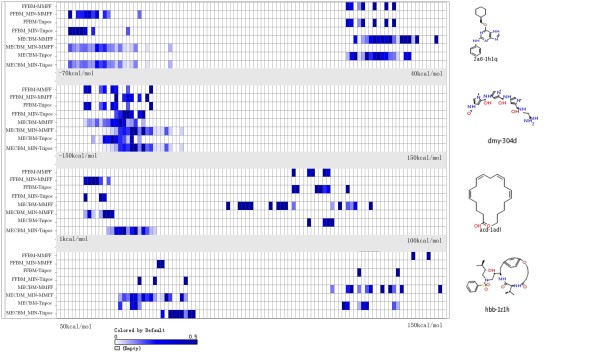
**Heat map showing the energies (in kcal/mol) of conformers obtained by the same test protocols used in Figure 3**. The scale of each molecule panel covers the full range of energy encountered across all conformations within the energy threshold window (20 kcal/mol above the global lowest energy identified by each test run). The intensity of the cell colour is proportional to the fractions of conformations that fall in each bin.

An ideal conformational search method must cast a wide net over the potential energy landscape and sample as broad a range of molecular geometries as possible [38]. Since the RMSD distribution does not provide full information on the diversity of conformational ensembles and can't fully quantize the coverage of low-energy conformational space, two other metrics were used to analyze the conformations, one is the conformation energy and the other is the gyration radius of conformation. To address this question, four representative molecules (including drug-like molecules, linear molecule, macro-cyclic molecule) with 5, 10, 15 and 20 rotatable bonds respectively, were selected from the dataset to be assessed. The distribution of gyration radius and the conformational energy for the four conformation ensembles were presented in the form of heat maps in Figures 3 and 4 respectively (for more representative molecules see Figures A1, A2, A3, A4, A5 and A6 in the Additional File 3), it should be noted that only conformations within 20 kcal/mol of the global lowest energy identified by each method were included for analysis in this work. For clarity, all heat maps are scaled according to the range of the energy/gyration radius of all conformations for each specific molecule. Figure 3 and Additional File 3 (A1, A2 and A3)  reveal that MECBM has a bias in favor of geometrically more extensive conformations and spans a relatively wider range of molecular radius of gyration than FFBM, especially when the molecule is more flexible. From Figures 4, and Additional File 3 (A4, A5 and A6) we can find a trend that the conformations generated by MECBM tend to stand on the lower states of energy spectrum. The difference of the conformations for the compact and relatively rigid molecule, like 2a6-1h1q, appears to be inconspicuous and some conformers out of the ensemble even tend to stand on higher energy states. In this case, an additional energy minimization refinement can drive them to lower energy space. Moreover, the size of the lower energy conformations set generated by MECBM is apparently larger with energies covering a broader region of the energy scale on the heat map, which suggests that the searching engine of the MECBM can greatly expand the range of conformations sampled and cover much broader spectrums of energies and geometric sizes than FFBM. The phenomenon becomes increasingly pronounced as the flexibility of molecule increases, which confirms the previous observation that flexible molecules often exhibit several different conformations but with nearly equal energy values[28], that is to say, Cyndi can dig out more diverse conformers within certain range of energy scale.

The efficiency of MECBM can be measured by the average CPU time consumed for calculating per molecule. The total numbers of unique conformations generated by MECBM is more than 5 times larger than the one generated by FFBM, while the total computational cost increases by only one third (Table 1). The significant increase of the total number of conformations generated by MECBM methods can be considered as a result of the expanded optimal solutions of *Pareto *frontier with multiple-objective optimization. Furthermore, the conformational search methods in Cyndi are tremendously faster but has a comparable conformational generation quality (Figure 2) in comparison with any method in MacroModel. As shown in Table 1, it takes only 0.3-0.4 seconds to deal with per molecule by using Cyndi, but it takes 41.7-132 seconds to do the same job by using MacroModel. On the other hand, the average sizes of the conformational ensembles for each molecule generated by the methods in MacroModel are remarkably larger than the ones generated by the methods in Cyndi, especially for MCMM and torsional/low-mode in MacroModel (523 for MCMM, 306 for torsional/low-mode, but only 34 for MECBM), and this may be consistent with the higher recovery rates and much more time consumption by MacroModel.

### Influence of Energy Minimization on Reproduction of the Bioactive Conformation by using FFBM and MECBM

In this work, the effect of the additional refinement by using energy minimization with different force fields has also been explored. As expected, post-refinement with minimization relatively improves the number of conformations which can meet the energy threshold, and this is true for every method examined, especially for MECBM (as shown in Table 1). However, the increased number of conformations doesn't fully indicate that the diversity of conformation becomes more abundant. As shown in Figures 3 and Additional File 3 (A1, A2 and A3) the minimization seems to work worse when FFBM is used, because the distribution scale of its generated conformers' gyration radiuses becomes narrower and more concentrated, which indicates that the minimization reduces the diversity of conformation ensemble in the case of FFBM. On the contrary, the minimization seems to widen the distribution scale of conformers' gyration radius and therefore improve the diversity of the conformers when MECBM is adopted and the molecule is more flexible. As we know, the final geometric structures of molecules are dependent on their corresponding starting structures in energy minimization process. As mentioned above, the conformations generated with FFBM are lack of diversity when compared with MECBM, which may limit the geometric diversity of the final conformations after energy minimization. Additionally, as shown in Table 1, compared with the process of conformational sampling without energy minimization, the computational cost will be multiplied several times if the post minimization is performed.

**Figure 5:**
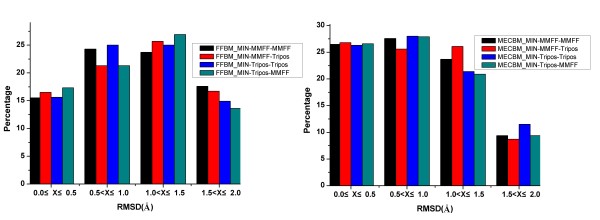
**The percentage of ligands reproduced within a particular RMSD from the bioactive conformation for two different methods with mixed-force fields mentioned in this work. (a) FFBM_MIN; (b) MECBM_MIN**. The names of the different protocols in this figure are defined as *M-C-F*, where *M *is the MECBM_MIN or FFBM_MIN, *C *is the force field type for calculating VDW and torsion energy terms employed both in these two methods and *F *is the force field type used in further energy minimization procedure.

To further examine the dependence on the force field type, the conformational sampling protocols with the above mentioned mixed force field strategy were performed and the corresponding distributions of RMSD between the bioactive conformations and the best fitting generated conformations were graphically summarized in Figure 5. It is pretty clear that using different force fields for conformation generation and post-refinement does not lead to an obvious enhancement of conformational generation ability of either MECBM or FFBM, and this therefore again confirmed that both MECBM and FFBM are insensitive to the force field type.

## Discussion

The results presented in this work indicate that MECBM can explore the low-energy conformational space more efficiently and more robustly than FFBM. Through integration with empirical geometric objectives, MECBM significantly increases the diversity of the generated conformational ensembles with the computational cost only increasing moderately. The enhanced diversity and conformational coverage of MECBM lead to a higher rate of reproduced bioactive conformers.

In the comparative study between Cyndi and MacroModel, the MCMM and torsional/low-mode of MacroModel adopting the systematic methods have the advantage of being able to retrieve more bioactive conformations than Cyndi which adopts the stochastic methods. However, the CPU time required by them is therfore remarkably larger than that of both MECBM and FFBM in Cyndi. Besides, in contrast with Cyndi, the tremendously large sizes of the conformational ensembles generated by the methods in MacroModel weaken their advantages and restrict their usage in the studies of relatively large number of molecules and large libraries. The above observations give us hope that the MECBM is promising for various modelling applications, especially for the high-throughput ones.

Moreover, a series of comparative analyses against different force fields reveal the conclusion that the qualities of conformation ensembles are non-sensitive to the force field type adopted neither in Cyndi nor in MacorModel. This confirmed the observation that it is not sufficient to minimize the raw conformations from any method and expect such improvements[38].

## Conclusions

This work provides a series of detailed comparative analysis of the conformational sampling tools available in our in-house soft package Cyndi and MacroModel. Regarding FFBM and MECBM methods in Cyndi, to realize a fair and reasonable comparison, we implemented MMFF94 as the alternative for force field to avoid the bias and then designed a series of protocols against a 742-molecule dataset to answer the questions regarding to i) the capability of reproducing bioactive conformation by using FFBM and MECBM respectively, ii) the influence of the geometric objectives on improving the reproduction of bioactive conformers, iii) the influence of the force fields on conformation generation, and iv) the effect of further energy minimization refinement with different force fields on the quality of conformation ensembles. The MECBM conformational models are distinctive for their enhanced diversity and conformational coverage, leading to a comparatively high rate of reproduced bioactive conformers. Due to the compromised mechanism of MOEA, MECBM can increase the number of conformer candidates remarkably but with nearly constant CPU time consumptions compared with FFBM.

The conformational generation is always a trade-off question between the sampling depth of conformational space and conformational costs with respect to the algorithm method used [35]. Therefore, the comparative analysis between Cyndi and MacroModel was focused on the balance between these two aspects. In terms of retrieving the bioactive conformations, the LMCS of MacroModel shows comparable performance to MECBM of Cyndi, while the MCMM and torsional/low-mode of MacroModel show higher recovery rates than that of MECBM. However, in terms of efficiency of the conformational sampling, the MECBM has the largest probability of discovering bioactive conformations at the fastest speed and this means that MECBM is the better choice for the generation of high-throughput generation, MCMM and torsional/low-mode approaches are the better choices for detailed studies of relatively fewer compounds or small libraries.

In summary, conformational sampling is a trade-off problem involving conformation energies and geometric diversity. By using different energy terms and geometric objectives, the MOGA based method can significantly expand the optimal solutions on *Pareto *frontier and increase the robustness of conformation sampling with more empirical criteria. As far as the conformation diversity and the reproduction of bioactive conformations are concerned, additional energy minimization is not necessary and may even reduce the diversity of conformational sampling. All the results guide us to explore more empirical criteria, which maybe play a pivotal role in the conformational generation of molecules.

## Authors' contributions

HL, XW and HJ conceived the study. FB, XL and HZ developed the methods, performed the analysis and drafted the manuscript. XL designed the validation protocol. HL, XL and JL reviewed and revised the manuscript. All authors have read and approved the final manuscript.

## Supplementary Material

Additional file 1 **The validation dataset used in this study**. The small molecules of the dataset are in Tripos mol2 format, and all these 742 molecules are stored in this file named "dateset.mol2". Click here for file

Additional file 2 **The validation protocol used in this study**. The validation protocol was developed with Pipeline Pilot v6.0 (Accelrys, Inc.). The input file is in Tripos mol2 format, and the output file is in HTML format which can be explored with the web browser. Click here for file

Additional file 3 **Heat maps for additional 30 molecules whose rotatable bonds vary from 1 to 25, 27 to 29, and 31 to 32**. This file is the complement to Figures 3 and 4, which consists of conformational information of additional 30 molecules to support the findings observed from Figure 3 and Figure 4. Click here for file
